# Treatment Failure and Post‐Artesunate Delayed Haemolysis in a Returned Traveller From Uganda With Partially Drug‐Resistant Severe *Plasmodium falciparum* Malaria

**DOI:** 10.5694/mja2.70136

**Published:** 2026-01-18

**Authors:** Jye Travis, Kate McCarthy, Paul Chapman, Lawrence Huang, Angelica Tan, Qin Cheng, Bridget E. Barber

**Affiliations:** ^1^ QIMR Berghofer Brisbane Queensland Australia; ^2^ Royal Brisbane and Women's Hospital Brisbane Queensland Australia; ^3^ Australian Defence Force Malaria and Infectious Disease Institute Brisbane Queensland Australia

**Keywords:** drug resistance, malaria, parasitic diseases

## Abstract

A man aged in his 40s, recently returned from Uganda, was hospitalised with *Plasmodium falciparum* malaria, with hyperparasitaemia of ~1.5 × 10^6^ parasites/μL (26%). He received intravenous artesunate followed by artemether–lumefantrine. However, parasite clearance was delayed, and despite a negative blood film following treatment, the patient was readmitted 3 weeks later with recurrent parasitaemia. Further testing for drug‐resistant phenotypes and genotypes demonstrated reduced susceptibility to lumefantrine, an A675V mutation in the *pfk13* gene and increased ring‐stage survival, consistent with partial artemisinin resistance. The case highlights the high risk of *P. falciparum* treatment failure in patients with hyperparasitaemia and partial drug resistance.

## Lessons From Practice

- Intravenous artesunate remains the treatment of choice for all patients with severe malaria and should be commenced without delay. The addition of intravenous quinine may be considered for patients with severe falciparum malaria from regions with established artemisinin resistance.
- Patients with severe *Plasmodium falciparum* hyperparasitaemia, particularly if the infection has been acquired in a region with antimalarial drug resistance, are at high risk of treatment failure, and a longer course of artemisinin combination treatment following initial intravenous artesunate should be considered.
- For patients with *P. falciparum* malaria returning from a region with antimalarial drug resistance, treatment with atovaquone–proguanil may be considered as an alternative to artemether–lumefantrine.
- All patients who have received intravenous artesunate should be monitored for post‐artesunate delayed‐onset haemolysis, with haematology checked at 7, 14, 21 and 28 days after initiation of artesunate therapy. Parasitaemia should also be monitored at these time points.

## 1 Clinical Record

**FIGURE 1:**
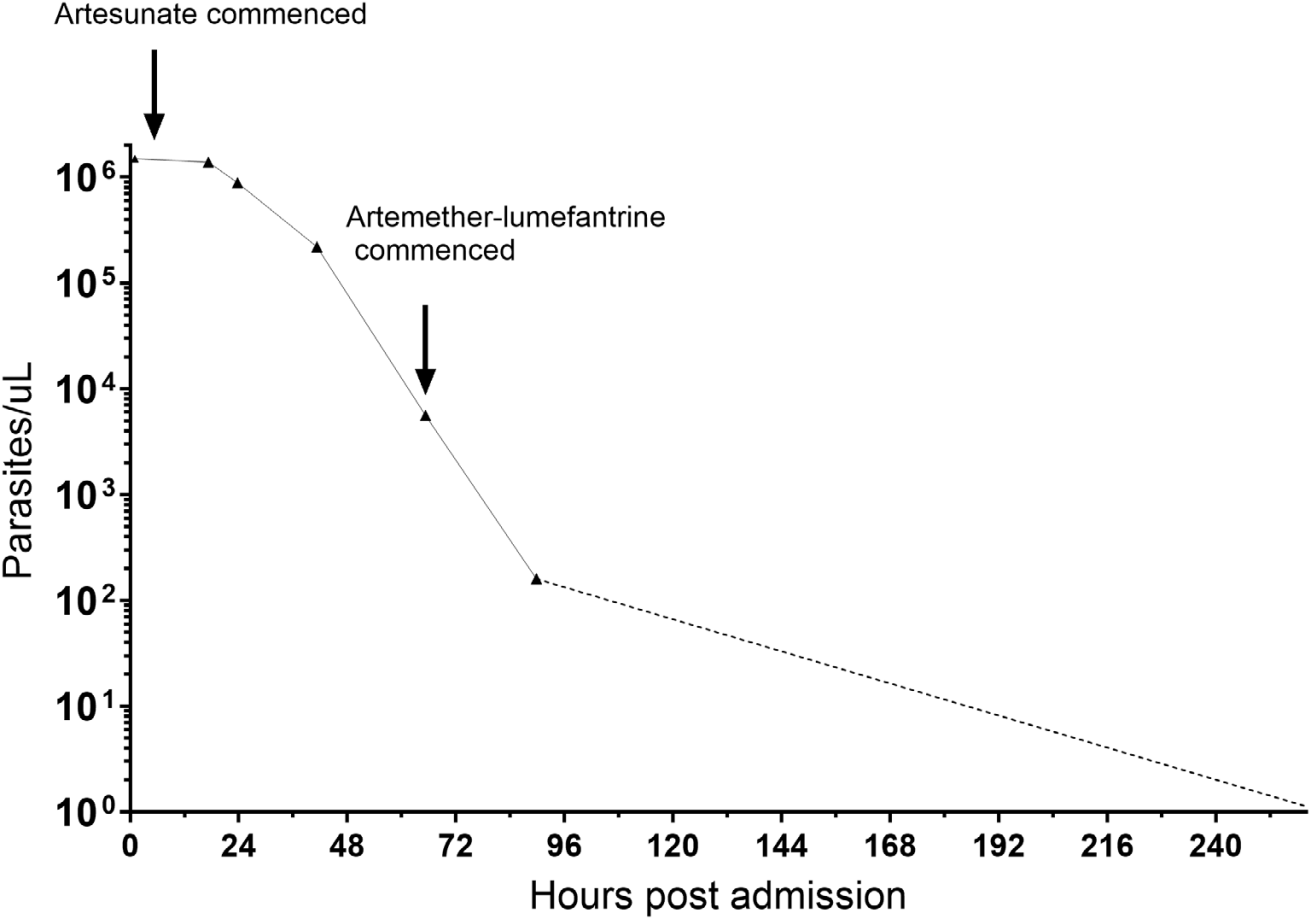
Parasite clearance after commencing intravenous artesunate during the first admission.

A man of European ancestry aged in his 40s presented to an emergency department in Brisbane, Australia, with a 2‐day history of headache, fever and back pain. Two weeks earlier, he had returned from a 3‐week trip to Uganda, without having taken malaria prophylaxis. On examination, his heart rate was 110 beats/min, temperature 39°C, blood pressure 110/80 mmHg and oxygen saturation 96% on room air. He was thrombocytopenic and had elevated liver transaminases (Table 1). A blood film demonstrated *Plasmodium falciparum*, ~1.5 × 10^6^ parasites/μL (26%). Intravenous artesunate was commenced, but parasite clearance was slow, with a parasite count decline of only 7.5% and 41% by 17 and 24 h, respectively (Table 1, Figure 1). He completed 4 days of intravenous artesunate. On day 5, his parasite count was 160 parasites/μL (< 0.1%); he was switched to oral artemether–lumefantrine and discharged. He completed a 3‐day course of artemether–lumefantrine on day 7. A blood film on day 11 was negative for malaria parasites; however, on day 18, his haemoglobin had declined to 108 g/L, which was a > 10% drop from the previous week, and, together with elevated lactate dehydrogenase, was consistent with post‐artesunate delayed‐onset haemolysis [1].

**TABLE 1:** Laboratory parameters and treatment administered during the first admission.

	Reference range	Day 1	Day 2 (AM)	Day 2 (PM)	Day 3	Day 4	Day 5	Day 11	Day 18
Time from commencement of artesunate (h)		0	17.28	23.72	41.25	65.23	89.80	264	
Parasite count, /μL (%)^a^		1,507,220 (26.4%)	1,394,160 (29.6%)	890,000 (18.8%)	220,000 (5.3%)	5600 (0.1%)	160 (< 0.1%)	Negative	Not done
Hb, g/L	135–180	158	138		123	130	143	135	108
WBC, ×10^9^/L	4.5–11.0	5.5	5.5	4.9	6.0	7.2	6.5	10.6	8.5
Platelets, ×10^9^/L	150–450	42	28	38	39	55	95	358	303
Creatinine, μmol/L	60–110	65	66		58	54	60	66	67
CRP, mg/L	< 5				306				
ALT, U/L	< 45	103	122		173	174	165		54
AST, U/L	< 35	93	115		148	102	76		69
ALP, U/L	30–110	78	76		101	101	118		
GGT, U/L	< 55	173	159		146	116	124	228	98
Bilirubin, μmol/L	< 20	50	23		19	15	12	26	37
LD, U/L	120–250	616	680		703	506	411	600	1350
Reticulocytes, ×10^9^/L	25–120								363
Blood cultures		Negative	Negative						
Treatment administered^b^		IV artesunate,^c^ ceftriaxone	IV artesunate		IV artesunate	IV artesunate	AL^d^		

Abbreviations: AL, artemether–lumefantrine; ALP, alkaline phosphatase; ALT, alanine transaminase; AST, aspartate transaminase; CRP, C‐reactive protein; GGT, γ‐glutamyl transferase; Hb, haemoglobin; IV, intravenous; LD, lactate dehydrogenase; WBC, white blood cell count.

^a^ Parasite count measured by microscopy from thick/thin blood film.

^b^ Paracetamol was also administered throughout the admission for renoprotection.

^c^ Artesunate was given at a dose of 2.4 mg/kg, with five doses given at 0, 13, 25, 37 and 61 h.

^d^ Artemether–lumefantrine (20/120 mg/tablet) was administered as four tablets twice daily for 3 days, given with fatty food or full‐fat milk to enhance absorption.

The following week, 25 days after the initial admission, the patient was readmitted with recurrent fever. He was jaundiced, with a temperature of 38.3°C, heart rate 130 beats/min and blood pressure 107/56 mmHg. A blood film demonstrated recurrent *P. falciparum*, 100,000 parasites/μL (4.4%), and laboratory results were again consistent with haemolysis, with haemoglobin 73 g/L, lactate dehydrogenase 978 U/L and haptoglobin < 0.01 g/L (Supporting Information: Section 1). He was transfused packed red blood cells and commenced on intravenous artesunate, atovaquone–proguanil and meropenem (Supporting Information: Section 1). He made a good recovery and was discharged 3 days later.

During the first admission, the patient's parasite clearance half‐life—calculated using the Worldwide Antimalarial Resistance Network (WWARN) parasite clearance estimator [2]—was 19.4 h, which was consistent with delayed parasite clearance and indicated possible drug resistance. Therefore, at the time of the second admission, blood collected in an EDTA (ethylenediaminetetraacetic acid) tube was obtained for testing drug‐resistant phenotypes and genotypes. Parasites (CMTM‐0232) were isolated and cultured in vitro, and susceptibilities to seven antimalarial drugs were evaluated using the 72‐h [3H]‐hypoxanthine growth inhibition assay. The drug‐sensitive *P. falciparum* 3D7 and the chloroquine‐ and piperaquine‐resistant/artemisinin‐partially resistant VPA02 strain [3] were used as controls. CMTM‐0232 demonstrated susceptibility to dihydroartemisinin, artesunate, artemether, mefloquine, chloroquine and atovaquone, comparable to 3D7, but reduced susceptibility to lumefantrine, with the half‐maximal inhibitory concentration (IC_50_) values 2.52‐ and 5.67‐fold higher than those of 3D7 and VPA02, respectively (Supporting Information: Section 2). An A675V mutation in the propeller region of the *P. falciparum kelch13* (*pfk13*) gene, a validated marker of artemisinin partial resistance [4], was detected in CMTM‐0232, in addition to a ring‐stage survival rate of 1.33%, demonstrating genotypic and phenotypic evidence of artemisinin partial resistance (Supporting Information: Section 3).

## 2 Discussion

Here we describe a case of treatment failure in a returned traveller from Uganda who presented with severe falciparum malaria, with hyperparasitaemia of ~1.5 million parasites/μL (26%). Despite prompt treatment with intravenous artesunate, parasite clearance was slow, with a half‐life of 19.4 h, well above the 5‐h threshold associated with artemisinin partial resistance [4]. The patient received 4 days of intravenous artesunate, exceeding the minimum duration recommended by the World Health Organization (WHO) guidelines [5], and parasitaemia had declined to 160 parasites/μL (< 0.1%) by the time of switching to oral artemether–lumefantrine. Despite this, and a negative blood film at completion of artemether–lumefantrine, the patient was readmitted on day 25 with recrudescent parasitaemia. The parasite carried the PfK13 A675V mutation, which has been reported in Uganda at a prevalence > 20% [6] and is associated with delayed parasite clearance [4]. The increased ring‐stage survival rate (> 1%) observed for this parasite is also consistent with partial artemisinin resistance. In addition, we detected reduced susceptibility to lumefantrine, which has been reported in Uganda [7] and may also have contributed to the treatment failure. However, the major contributor to the treatment failure was likely the very high parasitaemia. Even with 4 days of intravenous artesunate followed by 3 days of artemether–lumefantrine, the combination of high parasite burden and partial resistance to artemisinin and lumefantrine created substantial risk of persisting sub‐microscopic parasitaemia and subsequent recrudescence. The presence of gametocytes at the time of recrudescence highlights that in endemic regions, such cases present a high risk of transmitting drug‐resistant parasites [8].

The optimum regimen for treatment of severe malaria acquired in regions with established artemisinin partial resistance is uncertain. Although intravenous artesunate remains the treatment of choice and should be given without delay, WHO and Australian guidelines now recommend that intravenous artesunate and quinine be used in combination for such cases [5, 9]. This treatment strategy may be considered particularly in the setting of more severe complications such as cerebral malaria. Additional strategies to reduce risk of treatment failure include longer courses of artemisinin combination treatment, or alternative combination treatments such as atovaquone–proguanil [9].

This case also highlights the risk of haemolysis following treatment with artesunate. Post‐artesunate delayed‐onset haemolysis is common in non‐immune returned travellers, particularly in those with high parasitaemias, and results from splenic clearance of once‐infected erythrocytes [1]. Post‐treatment monitoring is therefore essential, with haematology checked weekly for 4 weeks following initiation of artesunate treatment [9].

## Author Contributions

**Jye Travis:** investigation, writing – initial draft, writing – review and editing. **Kate McCarthy:** investigation, writing – review and editing. **Paul Chapman:** investigation, writing – review and editing. **Lawrence Huang:** investigation, writing – review and editing. **Angelica Tan:** investigation, writing – review and editing. **Qin Cheng:** investigation, supervision, writing – review and editing. **Bridget E. Barber:** investigation, writing – initial draft, supervision, writing – review and editing.

## Funding

Bridget E. Barber is supported by the National Health and Medical Research Council Investigator Grant (#2016792). The funder had no role in the planning, writing or publication of this work.

## Disclosure

Not commissioned, externally peer reviewed.

## Consent

The patient provided written consent for the publication.

## Conflicts of Interest

The authors declare no conflicts of interest.

## Supporting information


**Data S1:** mja270136‐sup‐0001‐Supinfo.pdf.

## Data Availability

All relevant data are included in the article and its Supporting Information.
