# Supplementary material for: Treatment Failure and Post‐Artesunate Delayed Haemolysis in a Returned Traveller From Uganda With Partially Drug‐Resistant Severe Plasmodium falciparum Malaria
Source: Med J Aust. 2026 Jan 18;224(1):e70136. doi: 10.5694/mja2.70136 (PMC12813300; doi:10.5694/mja2.70136)
Supplement: Supplementary file 1 — Data S1: mja270136‐sup‐0001‐Supinfo.pdf. [file MJA2-224-0-s001.pdf]

The Medical Journal of Australia

Supporting Information

Supplementary material

This appendix was part of the submitted manuscript and has been peer reviewed.
It is posted as supplied by the authors.

Appendix to: Travis J, McCarthy K, Chapman P, Huang L, Tan A, Cheng Q, Barber BE. Treatment failure and post-artesunate delayed haemolysis in a returned traveller from Uganda with partially drug-resistant severe *Plasmodium falciparum* malaria. *Med J Aust* 2025; doi: 10.5694/mja2.70136.

Section 1: Laboratory parameters and treatment administered during the second admission

	Reference range	Day 1	Day 2	Day 3	Day 4	Day 5	Day 11	Day 18	Day 25	Day 32	Day 39
Time from commencement of artesunate (hours)		0	16.50	39.50	42.28						
Parasite count, /µL (%)		100,000 (4.4%)	22,000 (0.9%)	320 (< 0.1%)[a]	Negative	Negative	Negative	Negative	Negative		
Hb, g/L	135–180	73	77	86	89	100	106	124	130	132	139
WBC, × 10^9/L	4.5–11.0	6.2	4.2	5.3	5.5	9.3	5.2	6.0	5.6	6.4	6.4
Platelets, × 10^9/L	150–450	138	110	120	171	274	245	333	257	259	281
Creatinine, µmol/L	60–110	60	60	51	51	63	70	68			
CRP, mg/L	< 5		167		132						
ALT, U/L	< 45	39	38	63	53	62	59	54			
AST, U/L	< 35	59	74	126	83	49	39	37			
ALP, U/L	30–110	54	39	63	69	80	69	75			
GGT, U/L	< 55	55	48	87	77	77	54	51			
Bilirubin, µmol/L	< 20	41	92	105	37	21	17	14			
LD, U/L	120–250	978	949	1292	1305	856	499	399			
Reticulocytes, × 10^9/L	25–120	373	223								
Haptoglobin, g/L	0.40–2.80		< 0.01			< 0.01	< 0.01	< 0.01	0.04	0.27	0.68
Blood cultures		Negative									
Treatment administered[b]		IV art, meropenem, AP, 1 unit RBCs	IV art, meropenem, AP, 3 units RBCs	IV art, AP	AP	AP					

Hb = haemoglobin; WBC = white blood cell count; CRP = C-reactive protein; ALT = alanine transaminase; AST = aspartate transaminase; ALP = alkaline phosphatase; GGT = gamma-glutamyl transferase; LD = lactate dehydrogenase; IV = intravenous; art = artesunate; AP = atovaquone–proguanil; RBCs = red blood cells.

[a]Gametocytes observed. [b]Paracetamol was also administered throughout the admission for renoprotection.

Section 2: Susceptibility of *P. falciparum* strains CMTM-0232, 3D7 and VPA02 to a panel of antimalarial drugs. (A) IC_{50} values from two independent hypoxanthine incorporation assays in which incorporation was determined in triplicates for each drug concentration. **(B)** Growth inhibition curves from which IC_{50} values were determined. For each experiment (EXP), antimalarial drugs have been split across 2 graphs to improve readability. Error bars represent standard error of the mean from triplicates. DHA = dihydroartemisinin; AS = artesunate; ARM = artemether; CQ = chloroquine; ATQ = atovaquone; LMF = lumefantrine; MQ = mefloquine.

A.

	**IC_{50} (nM)**								
	**CMTM-0232**			**3D7**			**VPA02**		
	Exp 1	Exp 2	**Average**	Exp 1	Exp 2	**Average**	Exp 1	Exp 2	**Average**
**Dihydroartemisinin**	1.72	2.75	**2.23**	2.21	3.74	**2.98**	2.63	1.92	**2.28**
**Artesunate**	2.01	3.81	**2.91**	2.21	5.91	**4.06**	3.12	3.74	**3.43**
**Artemether**	5.98	9.04	**7.51**	5.65	13.25	**9.45**	8.43	7.34	**7.88**
**Mefloquine**	37.42	32.28	**34.85**	28.16	35.78	**31.97**	16.89	17.31	**17.10**
**Lumefantrine**	289.40	312.20	**300.80**	108.10	130.80	**119.45**	58.97	47.13	**53.05**
**Chloroquine**	16.27	16.01	**16.14**	11.93	15.95	**13.94**	387.70	427.40	**407.55**
**Atovaquone**	0.45	0.32	**0.38**	0.89	1.24	**1.06**	1.29	1.22	**1.26**

Exp = experiment

B.

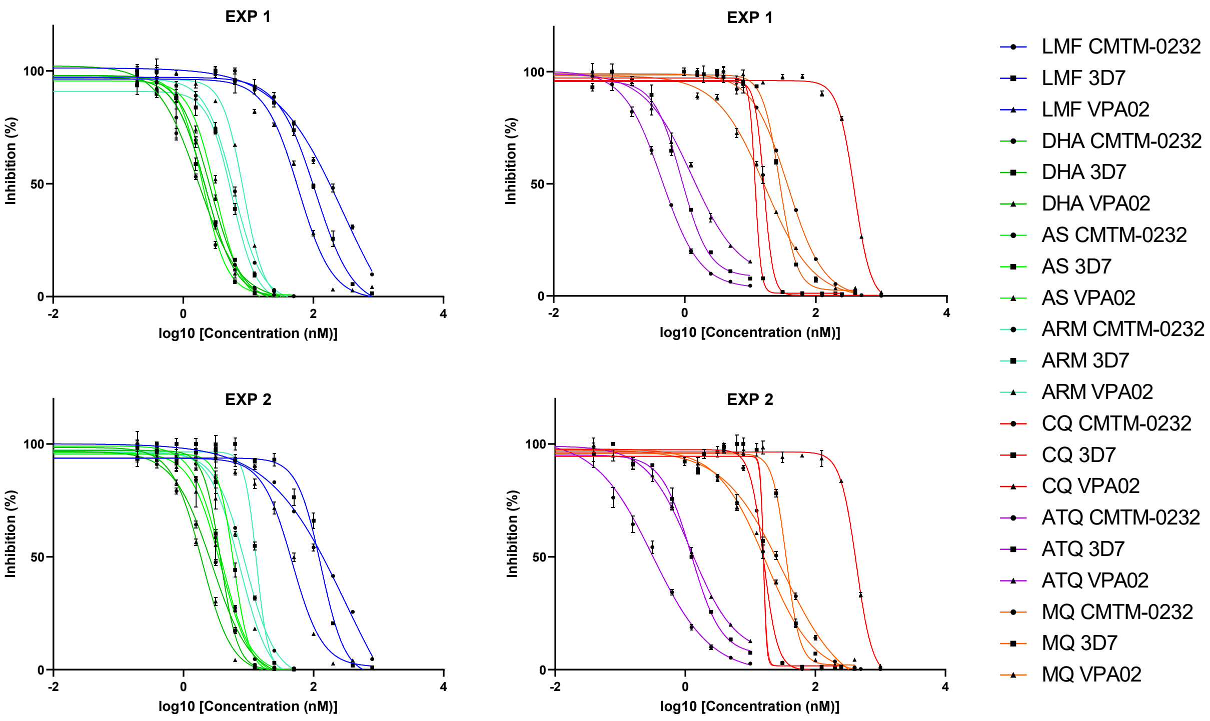

Section 3: Genotypic and phenotypic testing for artemisinin resistance. (A) DNA sequence and chromatogram alignments of fragments surrounding the nucleotide change encoding the A675V mutation between CMTM-0232 (sequences obtained using forward and reverse primers) and Pf3D7 (reference sequence from PlasmoDB). **(B)** Amino acid sequence alignment of the K13 propeller region from CMTM-0232 (sequences obtained using forward and reverse primers) and Pf3D7. **(C)** Ring stage survival rates of CMTM0232, 3D7 and VPA02. The rates were measured using the Worldwide Antimalarial Resistance Network (WWARN) ring stage survival assay protocol. DHA = dihydroartemisinin.

A.

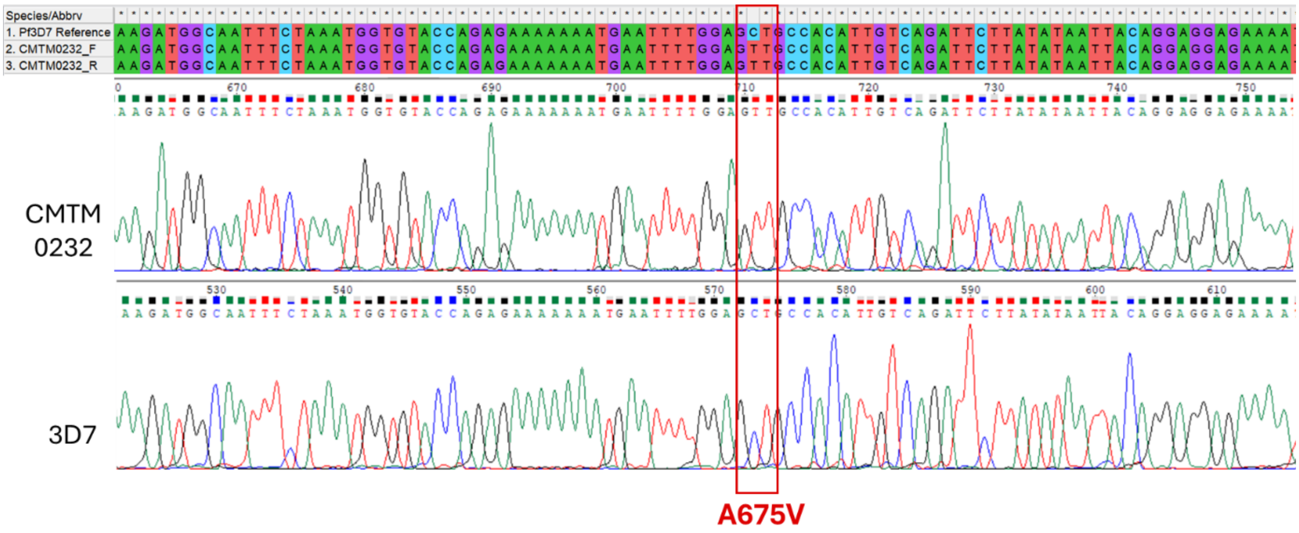

B.

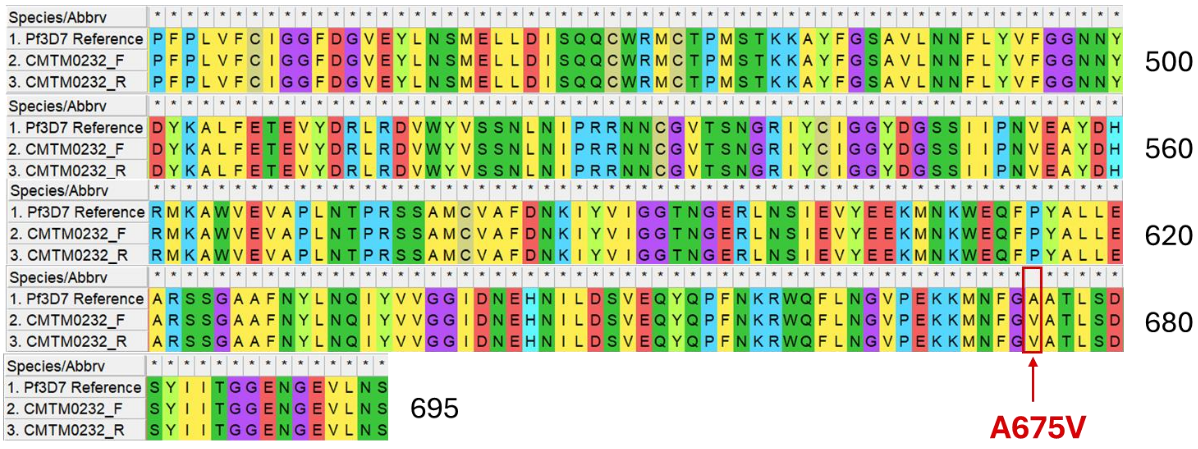

C.

Strain	Viable parasites per 10,000 RBC: Initial	Non-exposed	DHA-exposed	Growth rate (%)	Ring survival rate (%)
**CMTM-0232**	107	526	7	4.92	1.33
**3D7**	76	873	4	11.49	0.46
**VPA02**	108	342	6	3.17	1.75
